# New Agents for the Management of Advanced HER2-Positive Breast Cancer

**Published:** 2016-04-01

**Authors:** Melanie Royce, Karen Herold

**Affiliations:** University of New Mexico Comprehensive Cancer Center, Albuquerque, New Mexico, and Hoag Memorial Hospital Presbyterian, Newport Beach, California

Recent advances in the treatment of HER2-positive metastatic breast cancer have dramatically improved outcomes. Clinicians need to understand how to position the most effective new regimens and manage their unique side effects, speakers said at JADPRO Live at APSHO.

"The development of targeted biologics has been a major milestone in the treatment of HER2-positive metastatic breast cancer and has markedly improved survival," said Karen Herold, DNP, WHCNP-BC, FNP-BC, of Hoag Memorial Hospital Presbyterian, Newport Beach, California.

Melanie Royce, MD, PhD, of the University of New Mexico Comprehensive Cancer Center, Albuquerque, New Mexico, updated listeners on the key clinical trials that led to the approval of pertuzumab (Perjeta) and ado-trastuzumab emtansine (T-DM1; Kadcyla), two monoclonal antibodies that recently joined lapatinib (Tykerb) and trastuzumab (Herceptin) in the HER2-positive treatment arena, while Dr. Herold discussed how to monitor for and manage side effects associated with these new agents.

## T-DM1 APPROVAL

The agent T-DM1 links trastuzumab with the cytotoxic agent emtansine. It is indicated as a single agent in patients who progress during or after treatment with trastuzumab and a taxane.

The efficacy of T-DM1 was proven in the EMILIA study, which randomized 991 previously treated patients to receive T-DM1 or lapatinib/capecitabine (Verma et al., 2012). Median progression-free survival (PFS) was 9.6 months, vs. 6.4 months (hazard ratio [HR] = 0.65; *p* < .001), and median overall survival (OS) was 30.9 months, vs. 25.1 months (HR = 0.68; *p* < .001), respectively.

"The progression-free and overall survival curves separated and stayed separate. They’re the kind of survival curves you want to see," Dr. Royce commented. Adverse events were generally low grade, and patients were largely able to continue treatment after dose modifications.

"T-DM1 has therapeutic potential across a heterogeneous population of patients with advanced HER2-positive breast cancer," she noted.

## PERTUZUMAB APPROVAL

Pertuzumab targets the HER2 extracellular dimerization domain, allowing it to block ligand-dependent heterodimerization with multiple HER family members. Pertuzumab is indicated in combination with trastuzumab and docetaxel in the neoadjuvant setting (where it gained accelerated approval) and in untreated metastatic disease.

Pertuzumab received accelerated approval in the neoadjuvant setting based on the phase II NeoSphere trial, which evaluated four arms: docetaxel/trastuzumab; docetaxel/trastzumab/pertuzumab; trastuzumab/pertuzumab; and docetaxel/pertuzumab (Gianni et al., 2012).

Benefit was shown for combining the two antibodies with docetaxel. Rates of pathologic complete response (pCR) were 49% for patients receiving docetaxel/trastuzumab/pertuzumab compared with 18% to 31% for the other arms.

"Interestingly, 18% of patients achieved pCR without chemotherapy, just trastuzumab plus pertuzumab," Dr. Royce noted. "This means some patients with HER2-positive disease can eradicate tumor with antibody treatment alone, but we don’t know who these patients are yet and don’t want to deny the potential to cure them by withholding a known effective treatment, chemotherapy."

Pertuzumab is conditionally approved in the neoadjuvant setting, pending the results of the confirmatory phase III APHINITY trial.

Pertuzumab was approved for metastatic disease based on the phase III CLEOPATRA trial, which randomized 808 previously untreated patients to receive dual HER2 blockade with trastuzumab/pertuzumab/docetaxel or trastuzumab/docetaxel/placebo (Swain et al., 2015).

The addition of pertuzumab improved median PFS from 12.4 months to 18.7 months (HR = 0.68; *p* < .001) and median OS from 40.8 months to 56.5 months (HR = 0.68; *p* = .0002), yielding an unprecedented 16-month improvement in survival with dual HER2 blockade. "A survival time of almost 5 years is exceptionally long in this patient population," Dr. Royce observed.

Unfortunately, using the two antibodies, T-DM1 and pertuzumab in combination failed to provide additional benefit over trastuzumab/taxane in the phase III MARIANNE trial (Ellis et al., 2015). "So, trastuzumab/pertuzumab/docetaxel remains the first-line choice for metastatic breast cancer, with T-DM1 a second-line regimen," Dr. Royce reported.

## OLD STANDARDS ARE STILL EFFECTIVE

The new antibodies are often combined with chemotherapy for treatment of patients with hormone receptor-negative, HER2-positive disease, but they can also be used in patients with hormone receptor–positive, HER2-positive disease, although lapatinib plus letrozole also remains a standard regimen based on an improvement in PFS (but not OS) in the EGF30008 trial (Johnston et al., 2009). Dr. Royce reminded clinicians that, for patients who cannot take the newer drugs, older regimens can still be useful, including trastuzumab monotherapy; trastuzumab plus paclitaxel weekly or every 3 weeks; docetaxel plus trastuzumab; and carboplatin plus weekly paclitaxel and trastuzumab.

"In the metastatic setting, for second and third line, you don’t have to push the envelope to the point where the patient’s toxicity is worse than her disease," she remarked.

## EVALUATION OF PATIENTS WITH METASTATIC DISEASE

Dr. Herold discussed the workup of metastatic disease, recommending that in certain circumstances, patients should be retested for HER2 status (Figure). In newly diagnosed patients, if HER2 status on immunohistochemistry (IHC) staining is ≥ 2, the test is validated by fluorescence in situ hybridization (FISH). Patients are eligible for HER2-directed therapy if their FISH amplification is ≥ 2 or they are 3+ by IHC. In up to 16% of cases, initially HER2-negative patients will be HER2-positive upon metastasis and therefore eligible for anti-HER2 treatment.

**Figure:**
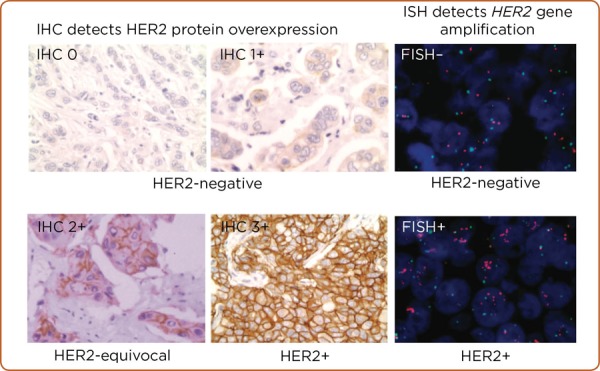
HER2 status of breast cancer cells determined by HER2 testing; The ASCO/CAP HER2 testing guidelines recommend that every case of primary, recurrent, or metastatic breast cancer be evaluated for HER2, estrogen-receptor, and progesterone-receptor status. Adapted from Wolff et al. (2013).

The initial workup for metastatic disease also includes a history and physical examination, complete blood cell count (CBC) and platelet count, liver function tests and assessment of alkaline phosphatase, positron-emission tomography/computed tomography (PET/CT), chest-diagnostic CT, bone scan, liver biopsy, and determination of hormone receptor status in the presumed metastatic site, she said.

Patients suspected of having a genetic predisposition should undergo genetic testing if it has not already been done at initial diagnosis. Primary care providers often fail to evaluate for familial syndromes, even when the history is indicative, noted Dr. Herold. "This is unfortunate," she added. "It’s imperative to evaluate patients for genetic risk syndromes when indicated, because we can do many things to keep them safe."

## MANAGING PATIENTS ON PERTUZUMAB

The best treatment for a patient with symptomatic HER2-positive/hormone receptor–positive recurrent disease is pertuzumab/trastuzumab/docetaxel, based on the CLEOPATRA study, Dr. Herold agreed. "We saw the benefits of this regimen in all subgroups, classified according to age, race, ethnicity, visceral vs. nonvisceral disease, HER2 status as determined by IHC or FISH, and previous exposure to neoadjuvant or adjuvant therapy."

Pertuzumab is given in a fixed dose and sequentially with the other two drugs. Docetaxel should follow pertuzumab and trastuzumab (which can be given in either order). For the first infusion, patients should be observed for 60 minutes after the loading dose of pertuzumab and before trastuzumab and docetaxel are given; for subsequent infusions, a 30-minute observation period is sufficient.

Before starting this regimen, it is recommended that patients undergo a multiple-gated acquisition (MUGA) scan. Premenopausal women should also be tested for pregnancy, since pertuzumab carries a black box warning for embryofetal toxicity.

Left ventricular ejection fraction (LVEF) should be measured at baseline and every 3 months. If it drops, trastuzumab and pertuzumab should be held, and LVEF should be rechecked in 3 weeks. Treatment can be restarted if LVEF recovers to > 40% or to 45% to 49%, with a < 10-point difference from baseline, she advised.

"Cardiotoxicity is a well-established adverse effect of trastuzumab, but treatment with trastuzumab has a life-extending or lifesaving potential, and it’s important to try to keep patients on it," Dr. Herold said.

The most common adverse reactions with the pertuzumab regimen are diarrhea, infusion reactions, febrile neutropenia, alopecia, nausea, fatigue, rash, and peripheral neuropathy. Mild-to-moderate diarrhea usually occurs in the first three cycles. Patients should be encouraged to hydrate aggressively, eat small meals, avoid lactose-containing products, and treat with loperamide at an initial dose of 4 mg followed by 2 mg every 4 hours or after every unformed stool, she advised.

## MANAGING PATIENTS ON T-DM1

Upon disease progression, the best treatment option for most patients is single-agent T-DM1. "Patients do quite well on this treatment, with relatively few side effects," according to Dr. Herold.

Treatment discontinuations are most common due to thrombocytopenia and increased aspartate transaminase. When platelet counts are < 25,000/µL, the drug should be held and resumed at a lower dose after counts have recovered to ≥ 75,000/µL or to baseline.

Recommended lab tests for patients taking T-DM1 include CBC, liver enzymes, and bilirubin levels at baseline and before each dose. A baseline LVEF level should be documented, and LVEF should be monitored every 3 months (or according to the patient history, signs, and symptoms).

Hemorrhage not related to platelet count, sometimes fatal, has been reported and could be associated with concurrent use of other drugs and supplements. "Check the other drugs that the patient is taking, such as fish oil. That’s important," Dr. Herold concluded.
